# AI-powered CT imaging for early detection of subclinical pulmonary fibrosis in antisynthetase syndrome: a new era in autoimmune lung diagnostics

**DOI:** 10.1097/MS9.0000000000003795

**Published:** 2025-08-28

**Authors:** Asiya Zubair, Ali Hamza, Maliha Khalid, Muhammad Talha, Aminath Waafira

**Affiliations:** a Jinnah Sindh Medical University, Karachi, Pakistan; b Mayo Hospital, Lahore, Pakistan; c Jinnah Sindh Medical University, Karachi, Pakistan; d King Edward Medical University, Lahore, Pakistan; e The Maldives National University, Malé, Maldives

**Keywords:** antisynthetase syndrome, artificial intelligence, CT imaging, pulmonary fibrosis

## Abstract

Antisynthetase syndrome (ASS), a rare autoimmune condition, often leads to subclinical pulmonary fibrosis (PF) that remains undetected until irreversible damage occurs. High-resolution computed tomography (HRCT) is pivotal in early PF detection, yet interpretation inconsistencies remain a challenge. Artificial intelligence (AI) and machine learning (ML) applications—such as multiple feature texture analysis, CALIPER, and deep learning segmentation tools—are revolutionizing lung imaging by minimizing observer variability, enhancing early diagnosis, and uncovering novel imaging biomarkers. While promising, the adoption of AI for rare disease diagnostics like ASS requires overcoming data limitations, bias, and validation hurdles. This letter calls for integrated genomic-imaging models, open-access repositories, and international data collaboration to effectively translate AI advancements into routine diagnostics for autoimmune lung disease.

*To the Editor,*

Antisynthetase syndrome (ASS) is an autoimmune condition that can cause Interstitial Lung Disease (ILD), characterized by inflammation or fibrosis within the interstitial space of the lungs. ILDs have over 200 different types, amongst which ASS induced ILD can lead to pulmonary fibrosis (PF), which is scarring of lung tissue. In most ASS patients, PF is subclinical, meaning that by the time cough or breathlessness appears, substantial damage has already occurred in the tissues. In the diagnosis of subclinical PF, high-resolution computed tomography (HRCT) plays a vital role, adding important information that cannot be determined from medical history and other diagnostic tests. Since accurate diagnosis has a large-scale dependence on HRCT, it is necessary to ensure that any errors in this technique are minimized. By integrating artificial intelligence into the diagnostic procedures, the efficacy of the treatment can be greatly increased^[1,2]^.

Machine learning methods have resulted in minimizing the inter-observer variations in the HRCT^[2]^. For instance, Multiple feature method is a lung texture analysis software that uses 26 mathematically derived image features to classify local CT regions into various categories (fibrosis, ground glass, honeycomb, etc.). In patients with IPF, the volume of groundglass reticular change using the adaptive, multiple-feature method has been associated with disease progression. Computer-Aided Lung Informatics for Pathology Evaluation and Rating (CALIPER) is an example of using machine learning to identify previously undetected biomarkers; pulmonary vessel volume identified by the software is associated with lung function decline across the ILD spectrum, independent of pulmonary hypertension^[3]^.

Recent studies highlight the use of Deep Learning (DL) models to detect underlying diseases. The CO-RADS AI system was successfully used for identifying patients with COVID-19 using chest CT scans^[4]^. The efficacy of these models in assessing and differentiating diseases is also supported by a retrospective study, which tested DL to segment thigh muscles and help quantitatively assess muscular inflammation of IIM through T2 mapping. This study also yielded conclusions in favor of utilizing artificial intelligence for more efficient treatment and prognosis of diseases^[5]^.

However, applying AI/ML for rare disease diagnosis such as ASS faces intrinsic validation challenges. Small, heterogeneous datasets render the training of algorithms prone to overfitting and bias. Multi-center collaborative efforts, open-access imaging/data repositories, and advanced data augmentation strategies have their groundbreaking features, but their limitations still have not been resolved completely. Besides, clinical implementation entails explainability as well as external validation—both usually under-addressed in early developmental technical reports^[6]^.

Concluding all this, AI-driven CT will bring drastic changes in the detection and management of pulmonary fibrosis in ASS, which might lead to early intervention and bespoke therapies. We insist on integrating genomic data into imaging algorithms and calling for international data-sharing initiatives to accelerate the translation of AI into real-world diagnostics for the detection of rare autoimmune lung diseases. Our work is in line with the TITAN Guidelines on the need for transparency in AI use in healthcare^[7]^.

## Data Availability

No data were generated for this manuscript.

## Peer and provenance statement

This article was not commissioned and was externally peer-reviewed.
